# Two-Step PEF Processing for Enhancing the Polyphenol Concentration and Decontaminating a Red Grape Juice

**DOI:** 10.3390/foods11040621

**Published:** 2022-02-21

**Authors:** Carlota Delso, Alejandro Berzosa, Jorge Sanz, Ignacio Álvarez, Javier Raso

**Affiliations:** Food Technology, Facultad de Veterinaria, Instituto Agroalimentario de Aragón-IA2, Universidad de Zaragoza-CITA, 50013 Zaragoza, Spain; carlotad@unizar.es (C.D.); aberzosa@unizar.es (A.B.); jsanzm@unizar.es (J.S.); ialvalan@unizar.es (I.Á.)

**Keywords:** red grape juice, pulsed electric fields, polyphenols, microbial control, novel food

## Abstract

This study’s aim is to evaluate Pulsed Electric Fields (PEF) technology as an alternative method for the processing of red grape juice. For this purpose, two PEF treatments were applied: first to grapes for polyphenol enrichment of the juice, and subsequently for microbial decontamination of the obtained juice. Juice obtained from PEF-treated grapes (5 kV/cm, 63.4 kJ/kg) had the polyphenol content 1.5-fold higher and colour intensity two times higher of control juices by spectrophotometric measurement (*p* ≤ 0.05). A subsequent decontamination treatment by PEF (17.5 kV/cm and 173.6 kJ/kg) achieved inactivation of the present microbiota (yeasts, moulds, and vegetative mesophilic bacteria) below detection level (<30 CFU/mL). Furthermore, PEF-treated juices were microbiologically stable up to 45 days, even at abusive refrigeration storage temperatures (10 °C). PEF juice quality and sensory characteristics were similar to a fresh juice; they were neither affected by the PEF decontamination treatment, nor by storage time and temperature. Results obtained in this study demonstrate the considerable potential of PEF for the production of a polyphenol-enriched and microbially stabilized red grape juice as a unique and sustainable alternative for the juice industry, while avoiding enzymatic and heat treatments.

## 1. Introduction 

Over the last decade, healthy foods have become one of the strongest trends among consumers, who want to adopt their diet as a lifestyle [1]. A diet based on fruit and vegetables has been associated with benefits for human health due to their high content in health-protective compounds [2]. Grapes are one of the most widely harvested fresh fruits worldwide, and their by-products (wine, grape juices, vinegar, herbal teas, etc.) are widely consumed [3] (Venkitasamy et al., 2019). Their singular organoleptic properties, high nutritional value, and versatility of processing make them one of the most highly appreciated fruits. They are rich in bioactive molecules including vitamins, minerals, carbohydrates, edible fibres, and phytochemicals. The most important phytochemicals in red grapes are polyphenols, which include anthocyanins, flavanoids, flavonols, stilbenes (resveratrol), and phenolic acids. These molecules have many useful biological properties: antioxidant, anti-inflammatory, anti-diabetic, antiplatelet, hepatoprotective, cardioprotective, and neuroprotective, many of which contribute to the prevention of certain serious diseases [4,5,6]. Certain grape compounds have recently been shown to possess properties that can work against different types of cancer [7,8], and/or which help decelerate cognitive decline [9] in vitro and in vivo.

Red grape juice, understood as a non-fermented and non-alcoholic beverage extracted from red grapes, is a healthy source of phenolic compounds. Red grape juice processing involves several operations aiming to obtain a high juice yield with a high concentration of polyphenols [10]. The most common method for red grape juice processing consists of hot pressing: heating grapes at 85 °C for 30 to 60 min, followed by enzymatic treatment with pectinolytic enzymes after having cooled the grape mass down to 50–60 °C [11,12]. One of the main objectives of this enzymatic treatment is the hydrolysis of the wall of grape skin cells with the purpose of facilitating the release of polyphenols located inside those cells. On the other hand, thermal processing at different intensities (depending on whether the product is distributed at room or chilled temperatures) is the traditional treatment applied to grape juice for microbial stabilization. The quality of red grape juice may be significantly affected by processing times and temperatures. Alternative processes with a reduced thermal load could help improve red grape quality while reducing the energetic cost of the process.

Pulsed Electric Fields (PEF) is a nonthermal technology that causes the formation of pores in the cytoplasmic membrane (electroporation), increasing its permeability and facilitating the transport of compounds in and out of cells. Due to its particular mechanism of action, PEF can be used for the improvement of mass transfer and microbial inactivation [13,14]. More recently the potential of PEF for enhancing the content of bioactive compounds in plant base foods have been observed [15].

Many studies have demonstrated the benefits of applying PEF to crushed and destemmed grapes with the purpose of causing irreversible electroporation of the cytoplasmic membrane of grape skin cells, which contain the majority of the compounds required for quality red wine. Just a few pulses at moderate electric field intensity (<4 kV/cm, <10 kJ/kg) permit a reduction of maceration times and/or improve the colour and concentration of polyphenolic compounds of red wine without impairing its sensorial attributes [16,17]. Typical maceration times applied in these studies range from 3 to 7 days [18,19,20]. More recently, Maza et al. [21] reported that red wines with a sufficient concentration of polyphenols could be obtained by treating the grapes with more intense PEF treatments (5 kV/cm, 53 kJ/kg), thereby reducing maceration time by 24 h. On the other hand, the efficacy of PEF for microbial decontamination of fruit juices including grape juice has been reported [14,22,23,24]. More intense PEF treatments are required for the electroporation of microbial cells (>10 kV/cm) and possible negative outcomes of PEF has been suggested produced by some electrochemical reactions such as the generation of ROSs or biomolecules denaturalization [25]. However, it has been reported that the treatment’s impact on physicochemical properties, nutritional values, and sensorial quality is minimal compared to untreated juices [26,27,28,29,30,31].

This study aims to develop a mild red grape juice processing method by applying PEF in two processing steps. First it is applied to grapes for the improvement of polyphenol extraction, and a further treatment is subsequently applied to the obtained juice for microbial decontamination.

## 2. Material and Methods

### 2.1. Grape Samples and Grape Juice Processing

**Figure 1:**
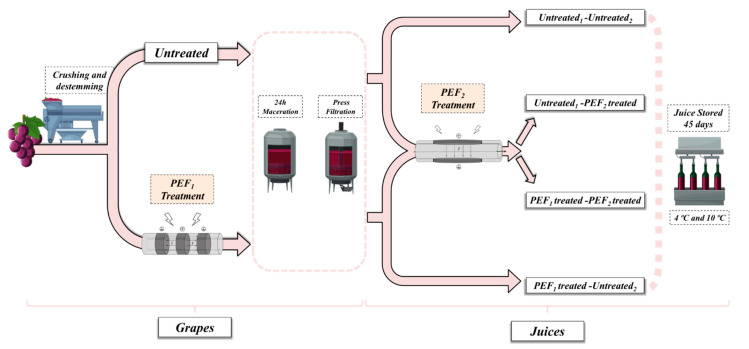
Simplified diagram of the experimental design and procedure accomplished in the present study.

Manually harvested *Grenache* grapes (*Vitis vinifera* L.) were obtained from the village of Fuendejalón (Aragón, Spain) in October 2020. Red grapes were crushed and destemmed with a Master E-10 destemmer (Enomundi, Zaragoza, Spain). Immediately after crushing, grapes were pumped (Rotor-MT, Bominox, Gerona, Spain) through a collinear treatment chamber, where they were subjected to the first PEF treatment (PEF_1_) aiming to improve polyphenol extraction. 25 kg of PEF_1_-treated and untreated grapes were distributed into steel tanks and stored for 24 h of maceration at 4 °C to prevent microbial growth. After maceration and after having pressed the solid parts of the grapes, the free-run and press juice thereby obtained were mixed. Juices obtained from untreated and PEF-treated grapes were treated by the second PEF (PEF_2_) for microbial decontamination. To evaluate the benefits of applying a PEF treatment for the improvement of polyphenol extraction and for microbial decontamination, four different samples were obtained: juice from untreated grapes without decontamination by PEF (Untreated_1_ + Untreated_2_) that was the control, juice from untreated grapes subsequently decontaminated by PEF (Untreated_1_ + PEF_2_), juice from PEF-treated grapes without decontamination by PEF (PEF_1_ + Untreated_2_) and juice from PEF-treated grapes decontaminated by PEF (PEF_1_ + PEF_2_). Two batches for conditions of around 2.5 L each were distributed in sterilized flasks of 250 mL and stored at 4 °C and 10 °C for 45 days. During storage, we performed physico-chemical, microbiological and sensory analyses. Figure 1 shows our experimental design and procedure.

### 2.2. PEF Treatments

In this study, a commercial PEF equipment (EPULSUS^®^-PM1-10 model; Energy Pulse System, Lisbon, Portugal) capable of applying monopolar square waveform pulses with a maximum voltage of 10 kV and a maximum current of 180 A and 3.5 kW of power was used. The actual voltage of each treatment was monitored by a high voltage probe (Tektronik, P6015A, Wilsonville, OR, USA) connected to an oscilloscope (Tektronik, TDS 220). In both PEF treatments, the products’ inlet and outlet temperatures were measured by means of a type K thermocouple (Ahlborn, Holzkirchen, Germany). Conductivity was measured by a conductivity probe (ALMEMO FYP641LFL1, Ahlborn) at 25 °C. Total specific energies (kJ/kg) have been calculated according to the following equation of pulse energy:

(1) W=VIτ  

*V* is the voltage applied (V), *I* the current intensity (A) and *τ* the pulse width (s). The specific energy (kJ/kg) was calculated by multiplying this energy per pulse (W) by the number of pulses and dividing it by the mass of the product. The oscilloscope measured the actual current intensity and voltage applied.

#### 2.2.1. PEF_1_-Treatment: Grapes

Crushed grapes with a conductivity of 1.4 mS/cm were pumped at 120 kg/h by a screw pump (Delta I-MV, Telangana, India) through a collinear PEF chamber with a residence time of 0.38 s. The chamber consisted of a central electrode connected to high voltage and two electrodes connected to ground. This configuration defines two treatment zones with a 2.0 cm gap and an inner diameter of 2.0 cm. Pulses of 40 µs width and 5 kV/cm were applied at a frequency of 120 Hz (45 pulses). The total specific energy was 63.4 kJ/kg, increasing the product’s temperature from 17 °C to 34 °C.

#### 2.2.2. PEF_2_-Treatment: Juice

Grape juice with a conductivity of 2.5 mS/cm was pre-tempered to 20 °C with a heating coil exchanger and pumped (peristaltic pump, BVP, Ismatec, Wertheim, Germany) at 10 L/h through a parallel electrode chamber of 0.4 cm gap with a residence time of 0.22 s. For microbial decontamination of the grape juice, 16 pulses of 10 µs of width and 17.5 kV/cm (173.6 kJ/kg) were applied at a frequency of 75 Hz. Outlet temperature of the sample after application of the PEF treatment was 65 ± 1 °C. The treated juice’s temperature was immediately reduced to under 10 °C in less than 5 s using a coil exchanger.

### 2.3. Microbiological Analysis

Microbial analysis was performed immediately after application of the PEF treatment (PEF_2_), and after 10, 15, 25, 30 and 45 days of storage at 4 °C and 10 °C. Samples were diluted in peptone water (Oxoid, Basingtok, Hampshire, UK), and 0.1 or 1 mL were plated onto different media. For yeast and mould quantification, samples were plated onto Potato Dextrose Agar (Oxoid) and incubated at 25 °C for 48 h and 5 days, respectively. For total aerobic mesophilic quantification, plating was counted on Plate Count Agar (Oxoid) incubated 24–72 h at 37 °C. Coliform quantification was conducted on Violet Red Bile Agar (Oxoid) incubated at 37 °C for 24 h.

The number of counted colonies corresponded with the number of viable cells expressed as colony-forming units per millilitre of juice (CFU/mL).

### 2.4. Physico-Chemical Analysis

#### 2.4.1. pH, °Brix, Total Acidity

pH, total soluble solids (°Brix), and total acidity were measured according to the methods prescribed by the OIV (Organization Internationale de la Vigne et du Vin) [32] by means of an electronic pHmeter (Crison Basic 20, Crison Instruments, Barcelona, Spain) and an Atago PR101 refractometer (Atago, Japan).

#### 2.4.2. Color Intensity (CI), CIELab Coordinates, Total Polyphenol Index (TPI) and Total Anthocyanin Content (TAC)

Samples were centrifuged for 15 min at 3000 rpm in an Eppendorf AG centrifuge (Eppendorf, Hamburg, Germany). All the analytical procedures for the determination of Color Intensity (CI), CIELab coordinates, Total Polyphenol Index (TPI) and Total Anthocyanin Content (TAC) were performed as described in Maza et al. (2019b) [33] using a Biochron Libra S12 spectrophotometer (Biochron, Cambridge, UK). The CIELab coordinates were calculated from the corresponding spectrophotometry measures (450 nm, 520 nm, 570 nm, 630 nm) using the MSCV software [34].

#### 2.4.3. Folin-Ciocalteu Index

Total phenolic content in the juices was determined by *Folin-Ciocalteu* index adapted from the reference method of the OIV [32]. 0.1 mL of samples previously diluted as required (1/3 or 1/5) were mixed with 5 mL of water. Subsequently, 0.5 mL of *Folin-Ciocalteau* reagent (Fisher Scientific, Hampton, VA, USA) were added, and then 2 mL of sodium carbonate solution (20% *m*/*v*), finally bringing the entire solution up to 10 mL with distilled water. After 30 min of incubation, absorbance at 750 nm was measured. A standard curve with Gallic acid (Sigma Aldrich, San Luis Obispo, CA, USA) was prepared within a concentration range of 80–400 µg/mL (R^2^ = 0.992). Results are expressed as µg of Gallic acid equivalents (GAE) per mL of juice.

#### 2.4.4. DPPH Assay: Antioxidant Activity

The antioxidant capacity of juice samples was evaluated by the reduction reaction of DPPH (2,2-diphenyl-1-picrylhydrazyl), turning their color from purple to yellow. Adequately diluted samples were mixed 1:1 with DPPH diluted in methanol (0.04 g/L). After 30 min of incubation under dark conditions, absorbance at 516 nm was measured using methanol plus water (1:1) as blank, and DPPH plus water (1:1) as control. A standard curve (R^2^ = 0.999) with different Trolox concentrations (0–10 µg/mL) diluted on methanol was established. Results of antioxidant capacity are expressed as µg of Trolox equivalents per mL of juice.

### 2.5. Determination of Phenolic Compounds by High-Performance Liquid Chromatography with Diode Array Detector (HPLC-DAD)

Analysis of individual phenolic compounds was performed by reverse-phase HPLC using an Agilent 1260 Infinity chromatograph with a diode-array detector (DAD). Grape juices were analysed at the onset (day 0) and after 45 days of storing at 4 and 10 °C. The protocol of the applied chromatographic conditions was previously reported by Portu et al. (2015) [35].

### 2.6. Sensory Analysis

Grape juices after 10 days of storing at 4 °C were sensorily evaluated in the sensory laboratory at the Pilot Plant of Food Science and Technology (University of Zaragoza). 16 selected panellists (7 men/9 women ranging from 22 to 52 years old) were distributed in individual booths and not informed about which samples would be tested. Before starting, clear instructions on how to proceed with the test were provided. With the objective of evaluating whether PEF treatment affected flavour and/or taste of red grape juices, two triangular discriminative analyses were performed using a completely randomized design. For this purpose, the compared samples were Untreated_1_ + PEF_2_ against PEF_1_ + PEF_2_ to evaluate the effect of the application of PEF to the grapes for improving polyphenol extraction, and PEF_1_ + Untreated_2_ against PEF_1_ + PEF_2_ to evaluate the effect of PEF aiming to achieve microbial decontamination. Samples were previously brought to room temperature and presented in opaque 10 mL plastic glasses. The tests were carried out under red light conditions to hinder the panellists from discriminating among juices by their colour.

### 2.7. Statistical Analysis

For each condition studied, the 2 independent batches obtained were distributed in different flasks. For each measurement 2 flask for each independent batch were analyzed in duplicate. Data are expressed as the mean ± the standard deviation of 4 measurement values. GraphPad Prism (Graph-Pad Software version 8.4.2, San Diego, CA, USA) was used for statistical analyses to evaluate the significance of differences among the mean values by student t-test or one-way analysis of variance (ANOVA) and Tukey test. Differences were considered significant at *p* ≤ 0.05. The significant difference for triangular tests was determined using statistical tables reported by Roessler et al. (1948).

## 3. Results and Discussion

### 3.1. Effect of Grape Electroporation by PEF on the Physico-Chemical Properties and the Individual Polyphenol Content of Juices

In order to detect the outcome of the electroporation of grape cells by PEF, Table 1 compares pH, °Brix, Acidity, Colour Intensity (CI), Total Polyphenol Index (TPI), Total Anthocyanin Content (TAC), total polyphenol content, and antioxidant capacity (DPPH) of juices obtained from untreated and PEF-treated grapes after 24 h of maceration at 4 °C. Maceration was conducted at this low temperature to prevent microbial growth. PEF treatment did not affect pH, °Brix and total acidity: no statistically significant differences were observed between the values of these parameters for the juices obtained from untreated and PEF-treated grapes (*p* > 0.05). These findings are in agreement with results reported by other authors who observed that these parameters were unaffected in must or red wine obtained from grapes treated by PEF, even under application of more intense conditions [17,36,37,38]. However, electroporation of the grape skin cells facilitated the release of the polyphenols located in the cytoplasm; consequently, CI, TPI, TAC, total polyphenols, and antioxidant capacity of the juice obtained from PEF-treated grapes were significantly higher (*p* ≤ 0.05). CI and TAC were two times higher in the case of the juice obtained from grapes treated by PEF, and values for TPI and total polyphenols were 1.5-fold higher. The higher effectivity observed for anthocyanin extraction than for total polyphenols could be related to the fact that anthocyanins are the most water-soluble polyphenols in grapes. It is well known that the extraction during winemaking of polyphenols other than anthocyanins is slower, and that the presence of ethanol obtained during fermentation facilitates the extraction of fewer water-soluble polyphenols such as tannins [39,40]. Anthocyanins are the main polyphenols responsible for the colouration of red grape juice; consequently, the higher the concentration of anthocyanins, the higher the colour intensity of the juice obtained from PEF-treated grapes [41]. Leong et al. [18] investigated the effect of PEF operated at electric fields greater than 30 kV/cm with energy inputs ranging from 4.7 to 49.4 kJ/L on anthocyanin extraction and colour intensity of Merlot juice plus 4 days of cold maceration. Even with the most intense PEF treatment (41.5 kV/cm, 49.4 kJ/L) and 4 days of maceration, the enhancement of the colour intensity of Merlot juice (×1.3 times) was lower than that obtained in our study at lower-intensity PEF conditions (5 kV/cm, 63.4 kJ/kg, 24 h maceration). Although this lower effectiveness could be related to the thick multilayer skin of Merlot grape berry cells, our results seem to indicate that PEF treatments of such a high electric field are not required for effective electroporation of grape skin cells.

Moreno-Montoro et al. [42] reported that the average total polyphenol content in different commercial red grape juices (*n* = 9) was 1177 ± 370 mgGAE/L. On the other hand, values between 1275 and 1410 mgGAE/L for Brazilian commercial red grape juices (*n* = 65) and from 714.4 to 826.6 mgGAE/L for European ones (*n* = 25) were reported by Granato et al. [43]. Our results show that grape electroporation by PEF yields a red grape juice with a concentration of polyphenols (1434.3 ± 154.6 mgGAE/L) slightly higher than in commercial juices, thus avoiding the use of hot pressing and /or pectinolytic enzymes.

Table 2 shows the content of the main polyphenol families and of individual polyphenols in red grape juices obtained from untreated and PEF-treated grapes. Results indicate that malvidin 3-glucoside and peonidin 3-glucoside were the main anthocyanins in both juices. However, the concentration of each individual anthocyanin was higher in the juice obtained from grapes treated by PEF in all cases (*p* ≤ 0.05). Grape electroporation improved the extraction of individual anthocyanins between 1.5 and 8 fold. The higher effect was observed for dephidin 3-glucoside, which increased from 1.35 mg/L to 11.04 mg/ L, and for petudinin 3-glucoside, which increased from 3.24 mg/L to 17.26 mg/L. Responsible for colour stability, malvidin 3-glucoside stands out as the major individual anthocyanin present, with twice the concentration in the PEF-treated samples (198.53 mg/L). A similar concentration of this anthocyanin (114.63 mg/L) was found in a Vitis vinifera red grape juice obtained by heat extraction (≈ 50 °C) and subsequent pressing [44]. On the other hand, 4 individual anthocyanins (cyanidin 3-acetylglucoside, cyanidin 3-trans-p-coumaroylglucoside, petunidin 3-trans-p-coumaroylglucoside, and peonidin 3-trans-p-coumaroylglucoside) that went undetected in the juice obtained from untreated grapes were detected when grapes were treated by PEF. In Merlot juice obtained from PEF-treated grapes after 4 days of maceration, certain anthocyanins not detected in the juice obtained from untreated grapes were observed by Leong et al. [18]. However, those anthocyanins (petunidin 3-glc, delphinidin 3-cmgl and malvidin 3-trans-cmglec) were different from those found in our study. This effect could be a consequence of the different composition of individual anthocyanins in merlot grapes.

Concerning the extraction of the other families of polyphenols, results showed a behavior similar to that observed for the extraction of anthocyanins. Flavonol, flavanols, and non-flavonoid content was 2.5, 1.7 and 1.8 fold higher, respectively, in the juice obtained from grapes treated by PEF (*p* ≤ 0.05). The flavonol quercetin in its two conformations (glucuronide and glucoside) was the main polyphenol found in both juices. However, the concentration of this flavonol, which is regarded as one of the polyphenols with the highest antioxidant activity, was 2 times higher in the juice obtained from grapes treated by PEF (*p* ≤ 0.05). A similar increment was caused by PEF electroporation in the total of hydroxyxinnamic acids and total stilbenes (*p* ≤ 0.05). It is remarkable that PEF increased the concentration of the stilbenes *trans* and *cis-resveratrol* by 50% and 100% in the juice obtained from treated grapes (*p* ≤ 0.05). Resveratrol is the most widely studied phenolic compound due to its beneficial properties: cardioprotective, antioxidant, anticancer, antidiabetic, neuroprotective, and anti-ageing [45].

### 3.2. Decontamination of Red Grape Juices by Pulsed Electric Fields

Red grape juices from untreated and PEF-treated grapes were decontaminated by PEF with the purpose of extending their shelf life. It is well known that the irreversible electroporation of the cytoplasmic membrane of microbial cells leads to their inactivation. However, to induce electroporation in microbial cells, the electric field strength required is higher than that required to electroporate cells of vegetable tissues [46]. Electroporation is a dynamic phenomenon that depends on local transmembrane voltage, and there is a specific transmembrane voltage threshold for the manifestation of electroporation. Transmembrane potential generated in the cell membrane by an external electric field depends on cell size as well as on the intensity of the electric field strength [47]. Consequently, we increased the external electric field we applied to treat the grapes (5 kV/cm) to 17.5 kV/cm for microbial decontamination of the red grape juices.

**Figure 2:**
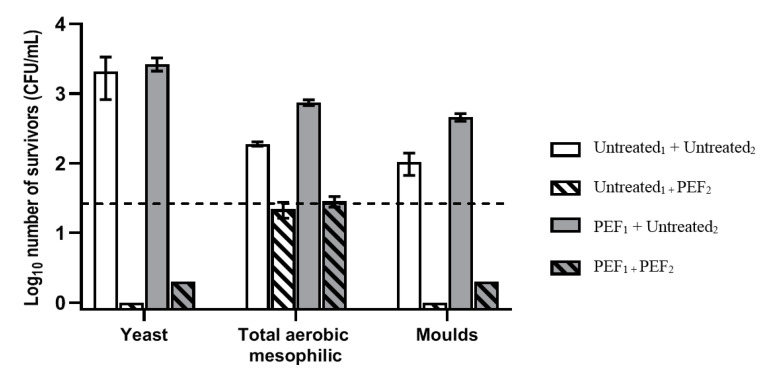
Impact of PEF treatment (17.5 kV/cm) on the number of survivors of yeast, total aerobic mesophilic bacteria and moulds in the grape juices. White bars (juice from untreated grapes without decontamination by PEF), white and dashed bars (juice from untreated grapes decontaminated by PEF), grey bars (juice from PEF-treated grapes without decontamination by PEF) and grey and dashed bars (juice from PEF treated-grapes decontaminated by PEF). Dashed line represents the quantification limit (30 CFU/mL ≈ 1.5 log_10_ CFU/mL).

The inactivation effects of PEF on the different groups of microorganisms that were contaminating the red grape juices obtained from untreated and PEF treated grapes are shown in Figure 2. Yeasts and moulds are the main type of microorganisms naturally present in fresh fruits, and, consequently, the main microorganism group present in fruit juices obtained from fresh fruits [48]. In the fruit juices obtained in this study, the highest concentration of microorganisms corresponded to yeast (2.5 × 10^3^ CFU/mL). No significant differences were detected in the concentration of yeasts in the fruit juices from untreated or PEF-treated grapes. Moulds and total aerobic mesophilic concentration were of the same order of magnitude (10^2^–10^3^ CFU/mL). Although the concentration of these two groups of microorganisms was lower to a statistically significant degree in grape juice obtained from untreated grapes, the differences are not of great practical consequence. Coliform bacteria were not detected in any of the samples analysed (data not shown).

Our selected PEF treatment achieved counts below the detection limit for yeasts (>3.0 Log_10_ reduction) and moulds (>2.0 Log_10_ reduction). The population of aerobic mesophilic bacteria was reduced by around 1.0 Log_10_ cycle. Microscope observations revealed that the survivor population of total aerobic mesophilic bacteria corresponded to sporulated forms that are not affected by PEF technology [49,50,51].

Microbial inactivation by the application of PEF has been widely investigated for purposes of pasteurization of different fruit juices such as orange, carrot, watermelon, and apple. Studies have shown a great effectivity of PEF, achieving up to 5.0 Log_10_ cycles reduction of the most concerning pathogens and spoilage microorganisms in juices under laboratory conditions [14,52,53,54]. However, the PEF parameters typically employed in these studies involved higher voltages (>25 kV/cm) and/or very long treatment times (>500 µs); these were thus treatments involving very high specific energies. Results reported on microbial inactivation in grape juices are thoroughly variable. Puértolas et al. [24] reported that a batch PEF treatment at 31 kV/cm (120 kJ/kg) caused an inactivation of around 4.0 Log_10_ cycles in the yeast population of *B. bruxellensis* and *S. cerevisiae* in artificially contaminated red grape must. Meanwhile, by increasing the PEF intensity, the indigenous population of yeast in a red grape juice was reduced by 3.9 Log_10_ cycles by applying PEF in 40 µs pulses at 80 kV/cm and 50 °C, while at 44 °C no significant microbial reduction was observed [55]. In our present study, the PEF treatment permitted to reduce the population of yeasts, moulds, and total aerobic mesophilic bacteria below the quantification limit by reducing the electric field to 17.5 kV/cm. This efficacy could be related to the use of a treatment chamber with parallel electrodes that permits a more homogenous distribution of the electric field as compared with a collinear configuration. Furthermore, the total specific energy applied (173.6 kJ/kg), corresponding with a temperature rise of the sample of around 40 °C during treatment, would have increased PEF inactivation thanks to a synergetic effect, as has been observed in combining PEF with non-lethal temperatures [14,56,57].

### 3.3. Changes in the Microbial Population and Quality Attributes of Grape Juices during Storing at 4 and 10 °C

After the application of a PEF treatment aiming to decontaminate red grape juices, we conducted a shelf-life study at 4 and 10 °C for 45 days. In order to ascertain the effect of PEF on microbial population and on quality attributes of red grape juice, this portion of the study featured samples obtained from untreated and treated grapes stored without a decontamination treatment as well as after the PEF decontamination treatment reported above.

#### 3.3.1. Change of Microbial Population of Grape Juices during Storage

Table 3 shows the evolution of the number of total aerobic mesophilic bacteria, yeasts, and moulds along storage time at 4 and 10 °C for the four samples of red grape juice.

For the two juices obtained from untreated and PEF-treated grapes that were not decontaminated by PEF, the yeast population was greater than 10^6^ CFU/mL after 10 storage days at 10 °C and 15 days at 4 °C. This increase in microbial population was accompanied by a decrease in the pH and °Brix of the juice, thus supporting the finding of juice spoilage (data not shown). Evolution of total aerobic mesophilic bacteria in these juices was similar to the evolution of yeasts, thereby indicating that the aerobic mesophilic bacteria present were mainly yeasts. No mould growth was observed in these juices, even after 45 days of storage. Timmermans et al. [58] observed an increase in yeasts and a decrease in moulds over time in untreated fruit smoothie stored at 4 and 7 °C. This effect was attributed either to the competition for nutrients, or to the fact that certain yeast species may inhibit mould growth [59].

Microbial population of samples treated by PEF was below quantification limits (<30 CFU/mL) for the three groups of microorganisms investigated after 45 days of storing at 4 and 10 °C. The fact that the population of total aerobic mesophilic bacteria that had survived the PEF treatment did not increase during storage even at 10 °C confirms that this population corresponded to bacterial spores unable to germinate in the low pH of grape juice. Our results disagree with those reported by Timmermans et al. [58] regarding the storing of an apple-strawberry-banana smoothie treated by PEF and stored at 4 and 7 °C. Those authors observed that moulds were visible in the PEF-treated smoothies after 14 days at 7 °C and after 18 days at 4 °C. This spoilage of the PEF treated-smoothie for moulds could be due to the presence in the raw material of mould spores that had a high resistance to PEF [60].

#### 3.3.2. Evolution of Quality Attributes of Grape Juices during Storage

In order to evaluate the effect of the PEF treatment in this study on quality attributes of red grape juices during storage at 4 and 10 °C, we analysed polyphenol composition, antioxidant activity, and colour in grape juices obtained from untreated and PEF-treated grapes microbially decontaminated by PEF after 45 days of storage. We did not conduct this study with the non-decontaminated grape juices because all of them were spoiled after 15 days of storage.

Total contents of the main families of polyphenols in the two fruit juices after storing at 4 and 10 °C are shown in Table 4. A significant reduction in the content of polyphenols of both juices during storage was observed for anthocyanins and flavonols, whereby this reduction was slightly higher in samples stored at 10 °C. Reduction of these two families of polyphenols was lower in the grape juice obtained from grapes treated by PEF. A reduction of 50% and 40% was observed for anthocyanin and flavonols, respectively, in the fruit juice obtained from untreated grapes, whereas in the juice from treated grapes the reduction was 27% and 10%. The decrease in content of anthocyanins and flavonols during storage is a widely observed phenomenon in wine ageing. In the case of anthocyanins, this decrease has generally been attributed to either degradation reactions or to a polymerization reaction with other compounds, specially flavanols, leading to the formation of more stable pigments. In the case of flavonols, the decrease has been associated with copigmentation reactions [61].

Concerning the other polyphenolic families, the reduction in concentration was lower than 10% at 4 °C for the juice obtained from untreated grapes. In the juice obtained from PEF-treated grapes, the concentration of flavanols and stilbenes was slightly higher after 45 days of incubation at 4 °C, whereas hydroxycinnamic acids only decreased by less than 2%.

On the other hand, total polyphenol content detected by Folin-Ciocalteu index (as mgGAE/L) prior to the application of the PEF decontamination treatment was around 56% higher in the juice obtained from grapes treated by PEF (*p* ≤ 0.05). After the application of the second PEF treatments and 45 days of storing at the two storage temperatures, total polyphenol values remained constant. Similar differences were found among the DPPH assay values (data not shown). Juices from PEF-treated grapes presented around 45% higher antioxidant capacity than untreated samples (*p* ≤ 0.05), and were neither affected by the PEF decontamination treatment, nor by storage time and temperature (*p* > 0.05). These results indicate that the behaviour of juice obtained from grapes treated by PEF during storage was similar to that of juice from untreated grapes. Moreover, the differences in total polyphenol content and in antioxidant activity detected after obtaining the juices were maintained after the application of the decontaminating PEF treatment and subsequent 45-day storage (*p* ≤ 0.05). These results confirm the observations made by certain authors that compared to other non-thermal treatments such as ultrasound [62,63]; PEF does not cause any degradation of polyphenols [28,64,65].

**Figure 3:**
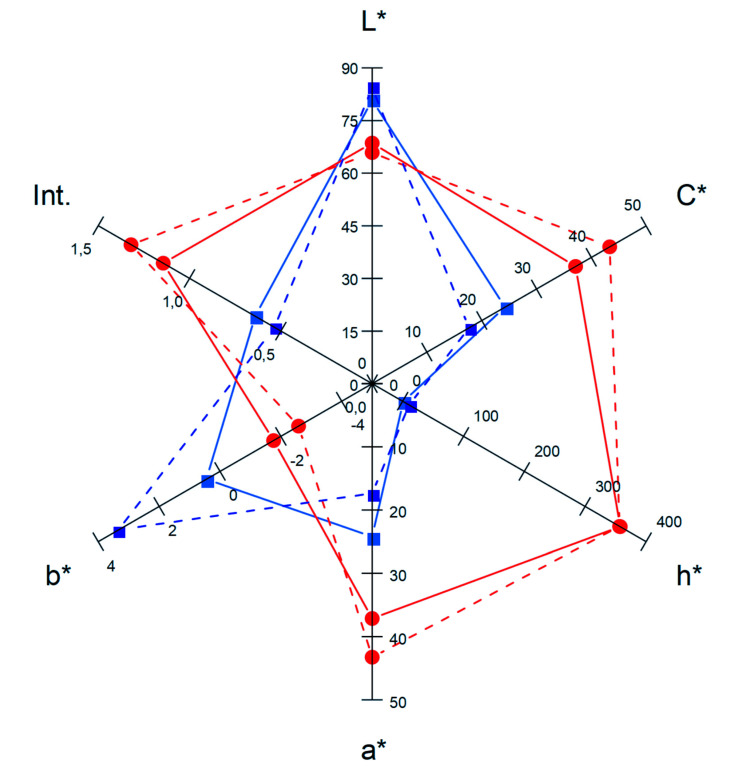
CIELab coordinates of juices obtained from untreated and PEF-treated grapes at the moment when they were obtained and after 45 days of storing at 4 °C. Juice obtained from untreated grapes (blue solid lines), juice obtained from PEF-treated grapes (red solid lines), juice obtained from untreated grapes after 45 days of storing at 4 °C (blue dashed lines), juice obtained from PEF-treated grapes after 45 days of storing at 4 °C (red dashed lines).

Colour, on the other hand, is an important quality attribute in fruit juices, and exerts a significant influence on consumer acceptance. As colour is an indicator of the changes that occur in juices during processing or storage, we compared colour intensity and CIELab coordinates of juices obtained from untreated and PEF-treated grapes. As no differences in these parameters were observed before and after the application of the PEF treatment aiming to decontaminate the juices (data not shown) (*p* > 0.05), Figure 3 compares the juices at the moment when they were obtained and after 45 days of storing at 4 °C. Juices stored for 45 days corresponded to those that were decontaminated by PEF. Colour intensity of the juice obtained from grapes treated by PEF was 2 times higher than control juice immediately after obtaining the juices, and even after the application of the PEF decontamination treatment (*p* ≤ 0.05). Similarly, to anthocyanin content, colour intensity decreased during storage, but the differences in colour intensity observed between grape juices obtained from untreated and PEF-treated grapes were maintained.

The evolution of CIELab parameters during storage was similar for grape juices obtained from untreated and PEF-treated grapes. Lightness (L*-value) and yellowness (b*-value) were higher for the juice obtained from untreated grapes (*p* ≤ 0.05). However, while the L*-value was not affected in the two juices by storing for 45 days, the b*-value decreased in both of them. Hue (h*-value), redness (a*-value), and chroma (C*-value) values were higher in the grape juice obtained from PEF-treated grapes (*p* ≤ 0.05). In this case, the h*-value did not change during storage, but the remaining coordinates decreased in both juices.

We therefore conclude that the application of a PEF treatment to grapes yields a grape juice with an intense colour and high C*, h* and a* values, corresponding to a saturated bright red colour. The differences (∆E) between L*, a* and b* coordinates among the juices obtained from untreated and PEF-treated grapes were greater than 3.0 units, which means that these differences can be easily perceived by consumers [66]. On the other hand, the differences between non-decontaminated and decontaminated juices would be imperceptible.

### 3.4. Sensory Analyses of Grape Juices

A sensory analysis was conducted to evaluate whether the PEF treatment modified the grape juices’ sensory properties. For this purpose, two independent triangular tests were carried out. In the first test, it was compared the red grape juices obtained from untreated and PEF-treated grapes and decontaminated by PEF. This test’s objective was to ascertain whether the PEF treatment applied to the grapes for the purpose of polyphenolic extraction resulted in a red grape juice that was sensorially different from control juice. Results obtained from the sensory analysis tests revealed that less than 45% of panellists were able to indicate which of the three presented samples was different. Therefore, neither the PEF treatment nor the higher concentration of polyphenols significantly affected (*p* ≤ 0.05) the sensory properties of the fruit juice obtained from PEF-treated grapes. Similar results were obtained in the second triangular test, in which we compared the juice with higher polyphenol content before and after the application of the PEF treatment aiming to achieve microbial decontamination. The PEF treatment applied at the highest electric field strength (17.5 kV/cm) thus did not lead to significant differences (*p* ≤ 0.05) in flavour and aroma of grape juice.

These findings thus show that PEF is a gentle technology with no negative impact on the sensory properties of grape juices. Our study confirms results obtained by other authors, who have reported that PEF did not have a negative sensory impact on the properties of juices [31,54,67].

## 4. Conclusions

The present study evaluated the potential of applying PEF technology in two different steps as a single alternative to the techniques currently applied to enhance polyphenol concentration and to achieve decontamination of red grape juices. The capacity of PEF for obtaining a polyphenol-enriched juice with 45-day microbiological stability and without negative impact on quality attributes and sensory properties was demonstrated. The current existence of PEF units capable of operating with clean, renewable energy sources and low energy consumption within the processing requirements of the juice industry supports the assertion that PEF technology may contribute to improving industry competitiveness for the production of healthy red grape juices. Optimization of industrial up-scaled conditions along with the applicability of PEF for both improving extraction and decontamination for other fruit juices are suggested for future studies.

## Figures and Tables

**Table 1:** Physico-chemical characterization of grape juices obtained from untreated and PEF-treated grapes after 24 h of maceration at 4 °C.

	Juice from Untreated Grapes	Juice from PEF-Treated Grapes
**pH**	3.52 ± 0.03 a	3.56 ± 0.04 a
**°Brix (g/100 g)** **^a^**	25.95 ± 0.14 a	26.35 ± 0.21 a
**Acidity (g/L)** **^b^**	3.95 ± 0.09 a	4.17 ± 0.08 a
CI * (A.U.)	8.46 ± 0.035 a	16.41 ± 0.09 b
TPI ** (A.U.)	28.65 ± 0.07 a	45.30 ± 0.28 b
TAC *** (mg/L) ^c^	242.39 ± 1.60 a	522.34 ± 24.14 b
**Total polyphenols (mgGAE/L) ^d^**	916.10 ± 13.10 a	1434.30 ± 154.60 b
**DPPH (ET** **μ** **g/mL) ^e^**	419.03 ± 19.05 a	604.28 ± 33.4 b

Values represent mean with standard deviation. Different letters within the same row indicate significant differences (*p* ≤ 0.05). * Colour Intensity. ** Total Polyphenol Index. *** Total Anthocyanin Content. A.U.: absorbance units. ^a^ Expressed as total soluble solids. ^b^ Expressed as tartaric acid. ^c^ Expressed as malvidin-3-glucoside. ^d^ Expressed as Gallic acid equivalents. ^e^ Expressed as Equivalent Trolox (μg/mL).

**Table 2:** Individual anthocyanin, flavanol, flavonol, and non-flavonoid content (mg/L) in grape juices obtained from untreated and PEF-treated grapes after 24 h of maceration at 4 °C.

**Anthocyanin Content (mg/L)**		
	**Juice from Untreated Grapes**	**Juice from PEF Treated Grapes**
delphinidin 3-glc	1.35 ± 0.06 a	11.04 ± 0.72 b
cyanidin 3-glc	3.06 ± 0.04 a	12.27 ± 0.77 b
petunidin 3-glc	3.24 ± 0.11 a	17.26 ± 0.76 b
peonidin 3-glc	28.11 ± 0.69 a	54.68 ± 1.89 b
malvidin 3-glc	85.25 ± 2.07 a	198.53 ± 6.33 b
delphinidin 3-acglc	0.79 ± 0.06 a	1.24 ± 0.04 b
cyanidin 3-acglc	n.d.	0.51 ± 0.04
petunidin 3-acglc	0.84 ± 0.02 a	1.39 ± 0.01 b
peonidin 3-acglc	0.51 ± 0.00 a	0.85 ± 0.02 b
malvidin 3-acglc	0.66 ± 0.01 a	1.61 ± 0.03 b
delphinidin 3-cmglc	2.38 ± 0.07 a	5.19 ± 0.14 b
cyanidin 3-cmglc	n.d.	0.63 ± 0.01
petunidin 3-cmglc	n.d.	0.60 ± 0.00
peonidin 3-cmglc	n.d.	0.77 ± 0.04
malvidin 3-*cis*-cmglc	0.50 ± 0.01 a	0.77 ± 0.01 b
malvidin 3-*trans*-cmglc	0.70 ± 0.03 a	2.19 ± 0.16 b
malvidin 3-cfglc	2.16 ± 0.14 a	10.07 ± 0.88 b
total anthocyanins	125.91 ± 2.86 a	309.45 ± 11.32 b
vitisin A	0.50 ± 0.01 a	0.70 ± 0.06 b
visitin B	0.69 ± 0.01 a	1.26 ± 0.01 b
**Flavonol Content (mg/L)**		
	**Juice from Untreated Grapes**	**Juice from PEF Treated Grapes**
myricetin 3-gal + glcu	4.69 ± 0.15 a	11.00 ± 0.04 b
myricetin 3-glc	3.06 ± 0.04 a	12.27 ± 0.77 b
quercetin 3-glcu	9.41 ± 0.08 a	19.53 ± 0.64 b
quercetin 3-glc	12.04 ± 0.54 a	27.45 ± 1.38 b
laricitrin 3-glc	3.45 ± 0.11 a	9.40 ± 0.49 b
kaempferol 3-gal	0.13 ± 0.04 a	0.56 ± 0.03 b
kaempferol 3-glc	0.45 ± 0.00 a	3.27 ± 0.06 b
isorhamnetin 3-gal	3.14 ± 0.18 a	7.40 ± 0.54 b
syringetin 3-glc	5.46 ± 0.15 a	11.12 ± 0.18
quercetin	0.40 ± 0.01 a	1.23 ± 0.04 b
laricitrin	n.d.	0.06 ± 0.02 b
total flavonols	46.88 ± 0.45 a	120.37 ± 5.43 b
**Flavanol Content (mg/L)**		
	**Juice from Untreated Grapes**	**Juice from PEF Treated Grapes**
epigallocatechin	6.17 ± 0.33 a	9.69 ± 0.35 b
Catechin	3.23 ± 0.36 a	10.12 ± 0.46 b
Epicatechin	4.96 ± 0.01 a	8.32 ± 1.45 b
Epicatechin gallate	0.53 ± 0.08 a	0.68 ± 0.06 b
Procianidin B1	6.95 ± 0.04 a	9.19 ± 0.32 b
Procianidin B2	1.10 ± 0.06 a	2.87 ± 0.29 b
total flavanols	25.18 ± 0.65 a	43.11 ± 1.47 b
**Non-Flavonoids Content (mg/L)**		
	**Juice from Untreated Grapes**	**Juice from PEF Treated Grapes**
Hydroxybenzoic acids		
gallic acid	n.d.	n.d.
Hydroxycinnamic acids		
caftaric acid	44.26 ± 0.69 a	77.45 ± 1.39 b
coutaric acid	8.88 ± 0.21 a	16.35 ± 0.13 b
fertaric acid	5.51 ± 0.16 a	6.83 ± 0.02 b
caffeic acid	1.29 ± 0.14 a	1.41 ± 0.04 a
coumaric acid	0.25 ± 0.00 a	0.23 ± 0.02 a
ferulic acid	1.75 ± 0.01 a	3.58 ± 0.15 b
total hydroxycinnamic acids	59.86 ± 1.18 a	103.75 ± 1.63 b
Stilbenes		
trans-piceid	0.78 ± 0.01 a	1.73 ± 0.13 b
cis-piceid	0.56 ± 0.01 a	0.83 ± 0.06 b
trans-resveratrol	0.12 ± 0.06 a	0.18 ± 0.02 a
cis-resveratrol	0.46 ± 0.01 a	0.99 ± 0.09 b
total stilbenes	1.95 ± 0.09 a	3.74 ± 0.09 b

Nomenclature abbreviations: glc, glucoside; acglc, acetylglucoside; cmglc, trans-p-coumaroylglucoside; cfglc, caffeoylglucoside; glcU, glucuronide; gal, galactoside; glc, glucoside; rut, rutinoside. All parameters are listed with mean and their standard deviation. For each row, values with different letters are significantly different between the samples (*p* ≤ 0.05).

**Table 3:** Change of microbial population (Log_10_ CFU/mL) along storage time in juice from untreated grapes without decontamination by PEF (Untreated_1 +_ Untreated_2_), juice from untreated grapes decontaminated by PEF (Untreated_1_ + PEF_2_), juice from PEF-treated grapes without decontamination by PEF (PEF1 + Untreated_2_), and juice from PEF-treated grapes decontaminated by PEF (PEF_1_ + PEF_2_).

**Total Aerobic Mesophilic Bacteria**						
		0 days	10 days	15 days	30 days	45 days
**Untreated_1_ + Untreated_2_**	4 °C	2.28 ± 0.03 ^a^	2.12 ± 0.16 ^a^	5.61 ± 0.29 ^b^	6.10 ± 0.05 ^b^	6.29 ± 0.05 ^b^
	10 °C		5.17 ± 0.25 ^b^	5.96 ± 0.31 ^bc^	6.80 ± 0.43 ^cd^	7.26 ± 0.03 ^d^
**Untreated_1_ + PEF_2_**	4 °C	<1.5	<1.5	<1.5	<1.5	<1.5
	10 °C		<1.5	<1.5	<1.5	<1.5
**PEF_1_ + Untreated_2_**	4 °C	2.87 ± 0.04 ^a^	4.17 ± 0.09 ^ab^	5.35 ± 0.01 ^bc^	5.92 ± 0.02 ^c^	5.86 ± 0.11 ^c^
	10 °C		6.42 ± 0.08 ^b^	6.78 ± 0.19 ^bc^	7.44 ± 0.28 ^c^	7.26 ± 0.17 ^bc^
**PEF_1_ + PEF_2_**	4 °C	<1.5	<1.5	<1.5	<1.5	<1.5
	10 °C		<1.5	<1.5	<1.5	<1.5
**Yeasts**						
		0 days	10 days	15 days	30 days	45 days
**Untreated_1_ + Untreated_2_**	4 °C	3.28 ± 0.28 ^a^	2.70 ± 0.26 ^a^	6.54 ± 0.09 ^a^	6.82 ± 0.02 ^b^	6.55 ± 0.11 ^b^
	10 °C		6.65 ± 0.07 ^b^	7.39 ± 0.02 ^b^	7.44 ± 0.06 ^b^	7.26 ± 0.09 ^b^
**Untreated_1_ + PEF_2_**	4 °C	n.d.	n.d.	<1.5	<1.5	<1.5
	10 °C		n.d.	<1.5	<1.5	<1.5
**PEF_1_ + Untreated_2_**	4 °C	3.42 ± 0.08 ^a^	3.58 ± 0.14 ^a^	6.39 ± 0.12 ^b^	6.60 ± 0.02 ^b^	6.62 ± 0.14 ^b^
	10 °C		6.65 ± 0.08 ^b^	7.35 ± 0.01 ^b^	7.52 ± 0.22 ^b^	7.23 ± 0.19 ^b^
**PEF_1_ + PEF_2_**	4 °C	n.d.	n.d.	<1.5	<1.5	n.d.
	10 °C		n.d.	<1.5	<1.5	n.d.
**Moulds**						
		0 days	10 days		30 days	45 days
**Untreated_1_ + Untreated_2_**	4 °C	2.00 ± 0.16 ^a^	2.31 ± 0.06 ^a^		2.15 ± 0.21 ^a^	2.27 ± 0.16 ^a^
	10 °C		2.21 ± 0.36 ^a^		2.07 ± 0.16 ^a^	2.26 ± 0.20 ^a^
**Untreated_1_ + PEF_2_**	4 °C	n.d.	n.d.		n.d.	n.d.
	10 °C		n.d.		n.d.	n.d.
**PEF_1_ + Untreated_2_**	4 °C	2.66 ± 0.05 ^a^	2.52 ± 0.21 ^a^		2.44 ± 0.14 ^a^	2.54 ± 0.21 ^a^
	10 °C		2.21 ± 0.29 ^a^		2.57 ± 0.12 ^a^	2.62 ± 0.11 ^a^
**PEF_1_ + PEF_2_**	4 °C	n.d.	n.d.		n.d.	n.d.
	10 °C		n.d.		n.d.	n.d.

n.d. = non detected. <1.5 log CFU/mL = below the quantification limit (30 CFU/mL). For each row, values with different letters are significantly different between the samples (*p* ≤ 0.05).

**Table 4:** Total content of anthocyanins, flavanols, flavonols, stilbenes, and hydroxycinnamic acids (mg/L) after 45 days of storing at 4 and 10 °C in grape juices obtained from untreated and PEF-treated grapes and subsequently decontaminated by PEF.

		Untreated_1_ + PEF_2_	PEF_1_ + PEF_2_
**Anthocyanins**	0 days	146.36 ± 3.34 a	340.30 ± 10.09 b
	45 days 4 °C	94.16 ± 14.06 a	267.86 ± 28.02 b
	45 days 10 °C	82.96 ± 8.92 a	204.88 ± 33.34 b
**Flavonols**	0 days	64.96 ± 1.92 a	139.59 ± 0.52 b
	45 days 4 °C	45.90 ± 0.40 a	125.08 ± 2.18 b
	45 days 10 °C	45.63 ± 6.39 a	105.03 ± 12.60 b
**Flavanols**	0 days	32.15 ± 4.06 a	48.65 ± 0.65 b
	45 days 4 °C	30.79 ± 1.84 a	51.77 ± 0.61 b
	45 days 10 °C	26.65 ± 3.32 a	44.93 ± 4.93 b
**Stilbenes**	0 days	2.76 ± 0.08 a	3.78 ± 0.06 b
	45 days 4 °C	2.51 ± 0.04 a	4.31 ± 0.03 b
	45 days 10 °C	2.40 ± 0.68 a	3.98 ± 0.03 b
**Hydroxycinnamic acids**	0 days	69.47 ± 0.48 a	110.64 ± 1.10 b
	45 days 4 °C	64.64 ± 3.84 a	108.69 ± 5.83 b
	45 days 10 °C	59.81 ± 9.66 a	105.28 ± 7.74 b

All parameters are listed with their mean and standard deviation. For each row, values with different letters are significantly different between the samples (*p* ≤ 0.05).

## Data Availability

Data is contained within the article.
